# Fe@N‐Graphene Nanoplatelet‐Embedded Carbon Nanofibers as Efficient Electrocatalysts for Oxygen Reduction Reaction

**DOI:** 10.1002/advs.201500205

**Published:** 2015-09-10

**Authors:** Young‐Wan Ju, Seonyoung Yoo, Changmin Kim, Seona Kim, In‐Yup Jeon, Jeeyoung Shin, Jong‐Beom Baek, Guntae Kim

**Affiliations:** ^1^ Department of Energy EngineeringUNISTUlsan689‐798Korea; ^2^ School of Material Science and EngineeringGeorgia Institute of TechnologyAtlantaGA30332USA; ^3^ Department of Mechanical EngineeringDong‐Eui UniversityBusan614‐714Korea

**Keywords:** electrospinning, energy conversion, Li‐air battery, oxygen reduction reaction, N‐doped graphene

## Abstract

**An activated carbon nanofiber (CNF) is prepared with incorporated Fe‐N‐doped graphene nanoplatelets (Fe@NGnPs),** via a novel and simple synthesis approach. The activated CNF–Fe@NGnP catalysts exhibit substantially improved activity for the oxygen reduction reaction compared to those of commercial carbon blacks and Pt/carbon catalysts.

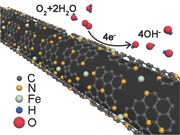

The development of highly active and stable catalysts for the oxygen reduction reaction (ORR) is an important task in the practical application of metal–air batteries and fuel cells.^1^, ^2^, ^3^, ^4^, ^5^ Although Pt and its alloys are considered the most effective electrocatalysts, having high intrinsic catalytic activity toward ORR,^6^, ^7^, ^8^, ^9^ they suffer from drawbacks of high cost and poor long‐term stability, which hinder the commercial expansion of Pt‐loaded batteries. In this regard, precious metal‐free catalysts such as carbon materials,^10^, ^11^, ^12^, ^13^ transition metal oxides,^14^, ^15^, ^16^, ^17^ and perovskite oxides^18^, ^19^ have been investigated as alternatives for the air electrode of metal‐air batteries.

Carbon materials have been widely used in recent decades for energy storage and/or conversion devices on the basis of their high electric conductivity, large surface area, chemical stability, and light weight. In particular, heteroatom‐embedded carbons such as N‐doped carbon,^10^, ^11^ graphene,^20^, ^22^ and carbon nanofiber (CNF)^16^ have been considered as alternatives to Pt‐based catalyst because of their high catalytic property and durability for the ORR. Nitrogen‐doped graphene has attracted tremendous attention as an ORR electrocatalyst while also serving as a support because of its high electric conductivity, high catalytic activity toward the ORR, and unique structure. Ozkan and co‐workers^13^ attributed the high electrochemical performance of heteroatom‐doped carbon to the improved interaction between carbon and heteroatoms, resulting in an enhanced electron‐donor property of the carbon. Baek and co‐workers fabricated heteroatom‐doped graphene nanoplatelets (GnPs) and reported superior electrochemical activity in an alkaline solution.^12^ Several research groups have also reported that additional additives such as Fe and Co enhanced the ORR activity in a heteroatom‐doped carbon catalyst.^13^, ^23^ In metal and nitrogen co‐doped carbon materials, the metal atoms are coordinated with the doped N atoms could provide active reaction sites for the electrochemical reaction.

Although the nature of the active ORR catalytic sites in such nitrogen–transition metal (Co and/or Fe)–carbon (N–M–C) catalysts continues to be at the center of an ongoing debate, it is clear that the ORR performance of N–M–C catalysts strongly depends on the type of nitrogen and transition‐metal precursors, the carbon support morphology, and the synthesis conditions.^24^

A schematic representation of the CNF fiber fabrication process based on a novel and simple synthesis approach is shown in **Figure** **1**. Along with material selection, the electrospinning technology, a unique method that can realize a composite fiber web using electrostatically repulsive force and a high‐voltage electric field, is important. The CNF web can be aligned along a one‐directional axis by electrospinning, which gives rise to fast electron transfer along the fiber axis. As a result, higher electrical conductivity of the aligned CNFs can be expected compared to that of normal carbon particles. Furthermore, this electrospun web can be transformed into a homogeneous composite CNF web via post treatment involving stabilization and carbonization.^25^ Finally, iron (Fe) and nitrogen (N) codoped GnPs (designated Fe@NGnPs) embedded in CNFs are enriched by activation in a CO_2_ atmosphere.

**Figure 1:**
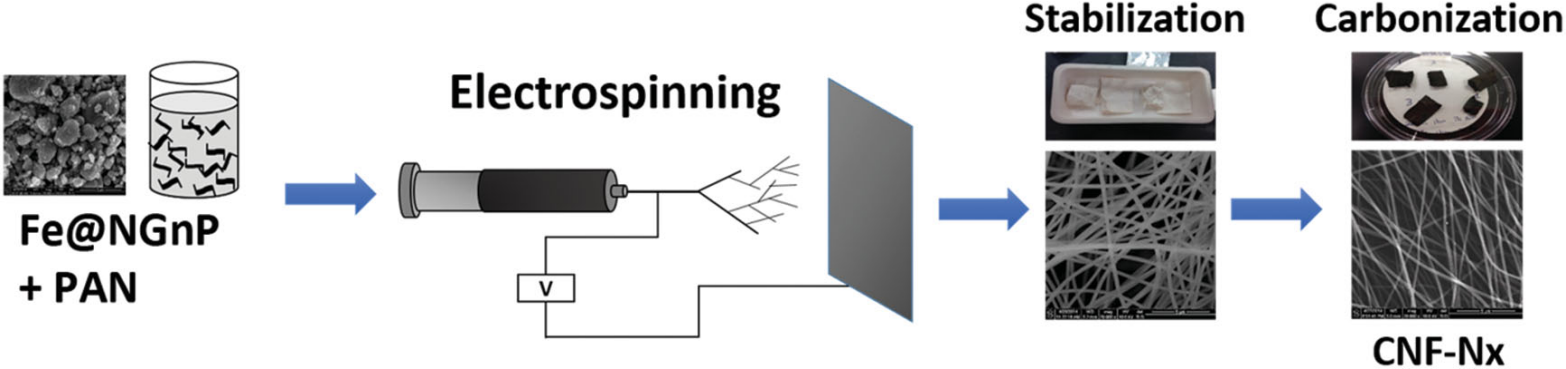
Schematic representation of carbon nanofiber with Fe@NGnPs by electrospinning method.

Here, we present a novel approach for the fabrication of precious metal‐free catalysts to be used as an effective ORR catalyst, which is prepared by electrospinning with a NGnP/PAN mixture solution and post‐treatment.^26^, ^27^, ^28^ This study demonstrates the optimization of the Fe@NGnP‐doped CNF microstructure via electrospinning, followed by a carbonization and activation process to maximize the performance. The electrochemical characteristics of the ORR catalyst, activated CNFs with Fe@NGnPs, are determined through rotating ring disk electrode (RRDE) measurements and hybrid Li–air cell experiments.

In order to confirm the fabrication of Fe@NGnPs, X‐ray diffraction (XRD) and X‐ray photoelectron spectra (XPS) analyses were performed. Figure S1a,b (Supporting Information) show the XRD pattern and XPS survey spectrum of Fe@NGnPs, respectively. The XRD pattern (Figure S1a, Supporting Information) reveals a broad peak at 24° (002) and a less intense peak at 43° (101). The broad diffraction peak indicates the existence of interlayer spacing of graphene, showing that the pristine graphite flakes were successfully converted into GnPs. The elemental composition of Fe@NGnPs by the XPS analysis only shows carbon, nitrogen, and oxygen peaks (Figure S1b, Supporting Information). Due to the sensitivity of heavy metallic atoms, the XPS could not detect iron. However, element mapping of transmission electron microscopy–energy dispersive X‐ray analysis (TEM–EDX) clearly shows the presence of iron in addition to carbon, nitrogen, and oxygen in the Fe@NGnPs (Figure S2, Supporting Information). The amount of iron in Fe@NGnPs is 3.81 at%.

Extended X‐ray absorption fine structure (EXAFS) measurements were also performed. X‐ray absorption near‐edge spectra (XANES) shows that the Fe@NGnPs has a similar oxidation state to that of Fe_3_O_4_, as shown in **Figure** **2**a. Compared with the structure of the Fe_3_O_4_ reference, the second shell and the third shell for the Fe@NGnPs are not detected in Figure 2b, because the Fe@NGnPs possesses an amorphous structure, implying the good distribution of Fe atoms. Figure 2c,d present the magnitude of the Fourier transformation and the fitted model curve, respectively. The quantitative results of Fe@NGnPs obtained from curve fitted radial distribution functions are listed in Table S1 (Supporting Information). Based on these analyses, it can be concluded that Fe‐ and N‐codoped graphene nanoplatelets (Fe@NGnPs) were successfully prepared.

**Figure 2:**
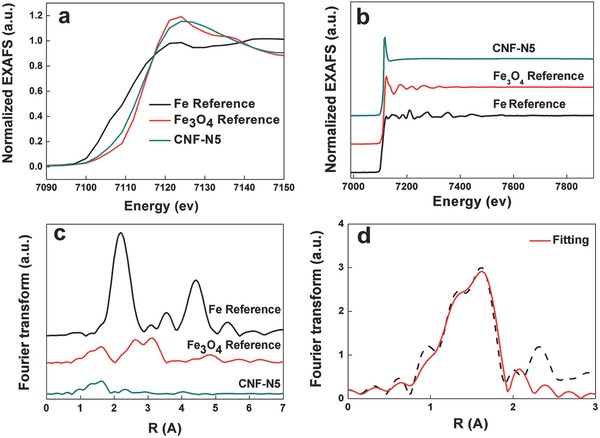
EXAFS spectra of CNF‐N5: a) XANES; b) normalized EXAFS; and c) Fourier transformed radial distribution. d) Fitted model curve.

The surface characterization of CNFs and CNF‐Fe@NGnPs is summarized in **Table** **1**. The 5 wt% Fe@NGnP‐embedded CNF(CNF‐N5) exhibited the largest specific surface area and total pore volume. The specific surface area and the total pore volume of CNF‐N5 were 17.445 m^2^ g^−1^ and 0.04961 cm^3^ g^−1^, while those of CNF‐N0 were 14.542 m^2^ g^−1^ and 0.04266 cm^3^ g^−1^, respectively.

**Table 1:** BET surface areas of the CNF–NGnPs

Sample	Surface area [m^2^ g^−1^]	Total pore volume [mL g^−1^]
CNF–N0	14.542	0.04266
CNF–N3	9.8126	0.03636
CNF–N5	17.445	0.04961
CNF–N10	13.493	0.03580

**Figure** **3**a,b present the morphology of CNF‐N5 and activated CNF‐N5 (denoted as Act‐CNF‐N5) observed by TEM. The CNF‐N5 sample shows a slightly rough surface due to the enriched Fe@NGnPs, which are well‐dispersed along the CNFs. In general, it is known that a charged jet forms a circle and a concentric annulus during an electrospinning process, and therefore it is speculated that the Fe@NGnP additive in the polymer solution is directed toward the exterior of the nanofibers by centrifugal force. In order to further increase the catalytic activity of CNF‐Fe@NGnPs for the ORR reaction, the FeNGnPs were exposed on the surface of the CNFs by activation in a CO_2_ atmosphere. Most of the surface of CNFs was covered with Fe@NGnPs after the activation process, as shown in Figure 3b. The enriched Fe@NGnPs on the surface of the CNFs could enhance both ORR and OER activity. The activation process also brings a significant increase in surface area, which is related with micropores developed during the activation process, as shown in Figure S4 (Supporting Information). The adsorption isotherms of Act‐CNF‐N5 (Figure S4a, Supporting Information) shows microporous adsorption at a low relative pressure (*P/P* ^0^ < 0.1) and hysteresis at a relative pressure *P*/*P* ^0^ = 0.5 or more, reflecting typical type II behavior showing microporous and mesoporous adsorption. Figure S4b (Supporting Information) shows the pore volume as a function of the pore size distribution based on the DFT method. The specific surface area and total pore volume is 201.42 m^2^ g^−1^ and 0.2096 cm^3^ g^−1^, respectively. The differential pore volume originated from increasement of the micropore volume along with activation process.

**Figure 3:**
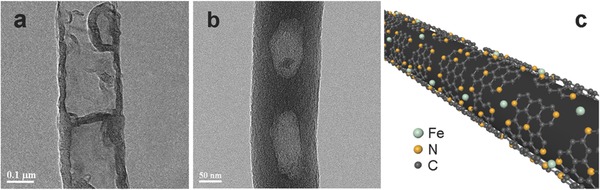
High resolution TEM images of a) CNF‐N5, b) Act‐CNF‐N5; and c) schematic diagram of activated CNFs with Fe@NGnPs.

The onset potential for the ORR is an important criterion to evaluate the activity of an electrocatalyst. Significant enhancement of the ORR catalytic activity in the CNF‐Fe@NGnP materials can be seen from the onset potential, as shown in **Figure** **4**a. The CNF−N5 composite appears to be a more active electrocatalyst than CNF‐N0, which has no Fe@NGnPs, considering the onset potentials and the limiting currents.

**Figure 4:**
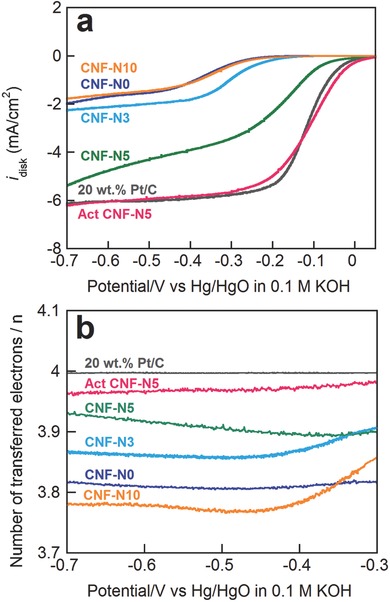
RRDE experiments data: a) disk current, and b) the number of transferred electrons (*n*) of ORR in 0.1 m KOH aqueous solution (scan rate = 10 mV s^−1^, rotation speed = 1600 rpm).

Above a potential range of −0.7 V, the electron transferred number (*n*) of CNF‐N5 is approximately 3.89–3.92, which is very close to that of 20 wt% Pt/C (3.99–4.0), whereas the *n* of PAN‐based carbon is 3.81 (Figure 4b). The results suggest that the ORR of CNF‐N5 is attributed dominantly to almost 4 electron reduction. Furthermore, CNF‐N5 has lower ring current density and peroxide yields than the other CNF materials, as shown in Figure S5a, b (Supporting Information). As the amount of Fe@NGnP in the CNFs is increased up to ≈5 wt%, the onset potential shifts to the positive direction. In addition, a higher disk current, a lower ring current, lower peroxide yield, and a nearly four‐electron reduction pathway (*n* ≈ 4) for the ORR are obtained.

The activation process could enhance the ORR in the mass transfer region due to the high surface area, the changes in morphology, and the catalytic property of the CNF‐Fe@NGnPs. Therefore, tailoring the surface via the activation process in a CO_2_ environment maximizes the electrochemical activities of CNF‐N5. As shown in Figure 4a, the onset potential for ORR of Act‐CNF‐N5 is enhanced to nearly that of 20 wt% Pt/C catalyst and is much superior to that of the non‐activated CNF‐N5. In addition, the limiting current of the Act‐CNF‐N5 sample was improved to −6.3 mA cm^−2^ compared to the value (−6.0 mA cm^−2^) of 20 wt% Pt/C. Act‐CNF‐N5 also exhibited the closest *n* (Figure 4b) and peroxide (Figure S5b, Supporting Information) to those of 20% Pt/C. Notably, the electron transfer number (*n*) of Act‐CNF‐N5 is nearly the same as that of 20 wt% Pt/C. This is related with enrichment of active sites such as pyridinic‐N and/or N–Fe on the surface by the activation, as shown in Figure 3c.

The electrochemical performance of Act‐CNF‐N5 was evaluated with a hybrid Li–air cell. **Figure** **5**a shows the first discharge–charge curve of 20 wt% Pt/C, carbon black (CB), and Act‐CNF‐N5 in 0.5 m LiOH + 1 m LiNO_3_ aqueous electrolyte at a current density of 0.5 mA cm^−2^. Discharge voltage plateaus are obtained at 2.95, 2.48, and 2.96 V vs. Li/Li^+^ for 20 wt% Pt/C, CB, and Act‐CNF‐N5, respectively. As expected from the ORR kinetics by the LSV results in Figure 4a, the discharge voltage of Act‐CNF‐N5 is comparable with that of Pt/C, due to the significantly enhanced electrocatalytic activity.

**Figure 5:**
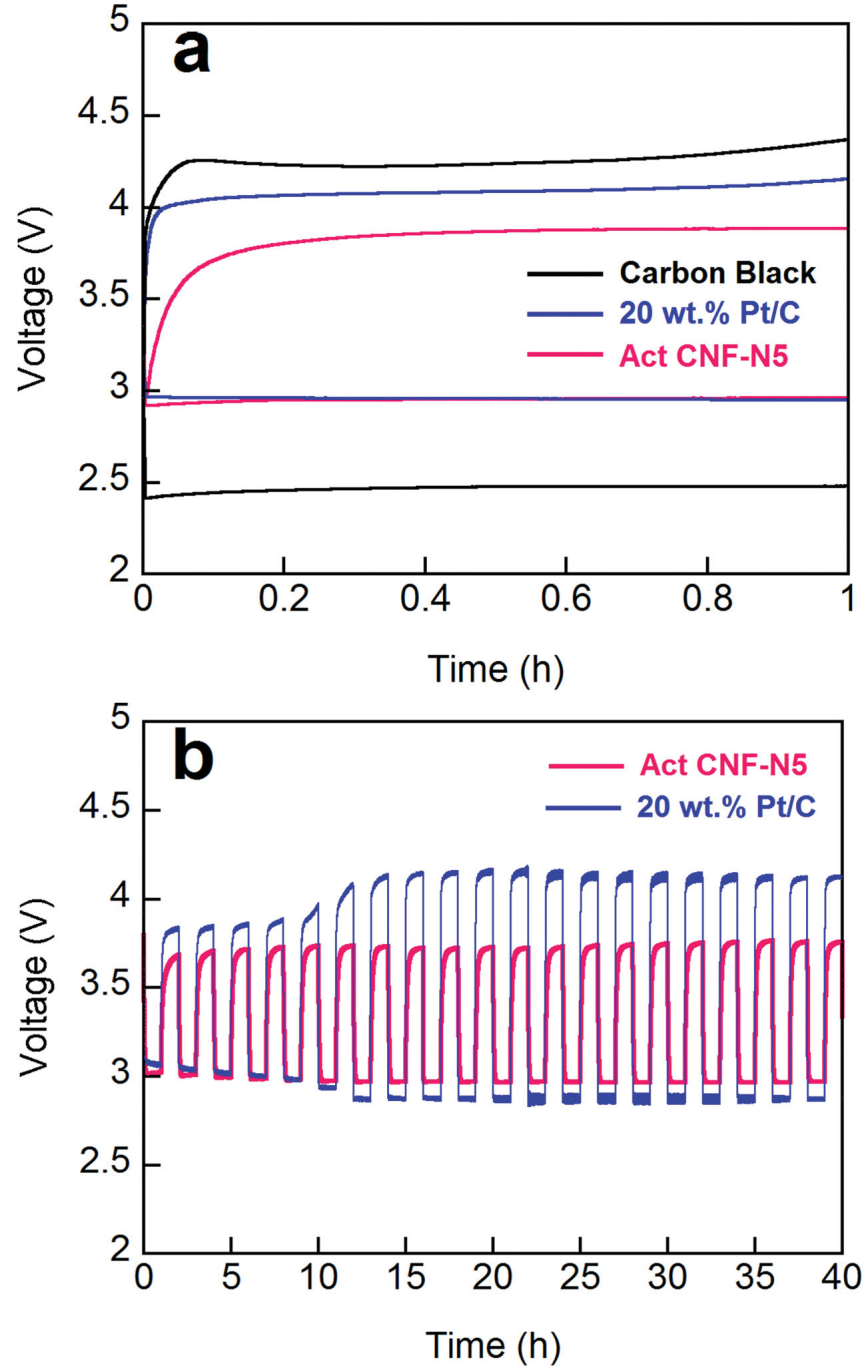
a) First charge–discharge curves of carbon black, 20 wt% Pt/C, and Act‐CNF‐N5 at a current density of 0.5 mA cm^−2^; and b) Charge–discharge cycle curves of Act CNF‐N5 and 20 wt% Pt/C at a current density of 0.1 mA cm^−2^ from 1 to 20 cycles.

The discharge profiles are investigated further at a higher current density in a range of 0.5 to 2.0 mA cm^−2^, as shown in Figure S6 (Supporting Information). At a current density of 0.5 mA cm^−2^, the discharge voltage of Act‐CNF‐N5 is almost the same as that of 20 wt% Pt/C. At a high current density of 2.0 mA cm^−2^, however, the discharge plateau of Act‐CNF‐N5 is even 0.17 V higher than that of 20 wt% Pt/C. It can therefore be confirmed that the ORR kinetics of Act‐CNF‐N5 are better than those of 20 wt% Pt/C even at higher current densities. Figure 5b presents the cycling performance of Act‐CNF‐N5 and 20 wt% Pt/C at a constant current density of 0.1 mA cm^−2^ in ambient air. The 20 wt% Pt/C exhibits gradual extension of voltage window after 10 h, while Act‐CNF‐N5 shows excellent cycling stability during 40 h without any degradation. The substantial improvement of Act‐CNF‐N5 can be explained by the four‐electron dominant pathway and higher catalytic activity for the ORR.^29^, ^30^

The change in surface morphology by activation can explain the outstanding electrocatalytic activity of Act‐CNF‐N5, which is comparable to that of a Pt/C catalyst, due possibly to the evolution of the surface functional groups. Figure S7a,b (Supporting Information) show XPS spectra of N 1s for CNF‐N5 and Act‐CNF‐N5, respectively. Three N species were present in the CNF‐Fe@NGnP composites, pyridinic‐N, pyrrolic‐N, and graphitic‐N. Xing et al. demonstrated that the carbon atom neighboring pyridinic‐N plays an important role in the ORR process and the location of pyridinic‐N can affect the catalytic efficiency of carbon materials.^28^ In addition, Dodelet et al. proposed that most of the Fe/N/C catalytic sites consist of an iron cation coordinated by four pyridinic‐N.^23^ The amount of pyridinic‐N is hence considered to be important with respect to the number of active catalytic sites, and the increase of pyridinic‐N after activation to 43.4% from 39.7% can partly explain the excellent performance of Act‐CNF‐N5.

In summary, we have investigated CNF incorporated Fe‐N‐codoped graphene nanoplatelets (Fe@NGnP) prepared using a novel and simple approach for the synthesis of non‐precious metal CNFs. A small amount of Fe@NGnPs coated on CNFs resulted in significant improvement of the catalytic activity toward the ORR without using precious Pt. The enhanced catalytic activity originated from enrichment of the functional groups along with CO_2_ activation. After activation, the 5 wt% Fe@NGnP‐embedded CNF (Act‐CNF‐N5) showed excellent performance in both ORR activity and charge–discharge behavior compared to commercial Pt/C. The Act‐CNF‐Fe@NGnP composite therefore can be considered a superior metal‐free electrocatalyst to Pt/C for the ORR in a metal–air battery.

## Experimental Section

*Preparation of Fe@NGnPs, CNF, and CNF‐FeNGnP Composite*: Fe@NGnPs were prepared by ball‐milling pristine graphite flakes in a planetary ball‐mill machine (Pulverisette 6, Fritsch) in the presence of nitrogen (N_2_). The pristine graphite (5.0 g, Alfa Aesar, natural graphite, 100 mesh (<150 μm), 99.9995% metals basis, Lot#14735) was placed into a stainless steel ball‐mill capsule (500 mL) containing stainless steel balls (500.0 g, diameter 5 mm). The capsule was sealed and charged with N_2_ (8 bar of cylinder pressure) after five charging–discharging cycles and then fixed in the planetary ball‐mill machine and agitated at 500 rpm for 48 h. The final product was freeze‐dried at −120 °C under a reduced pressure (0.05 mmHg) for 48 h The source of iron (Fe) is the stainless steel balls and ball‐mill capsules.^12^ PAN (Molecular weight = 150–000, Aldrich Chemical Co.) and *N,N*‐dimethylformamide (DMF, Alfa Aesar Co.) were used as a carbon source in the CNFs and a solvent, respectively. The PAN‐based fibers and Fe@NGnP‐embedded PAN‐based fibers were prepared by electrospinning using a 10 wt% PAN solution in DMF and a composite solution, which was prepared by mixing Fe@NGnPs in DMF and PAN solutions in DMF. For the Fe@NGnP‐dispersed solution, the Fe@NGnPs and DMF mixture was sonicated by a homogenizer for 30 min in order to completely disperse them prior to mixing with the PAN solution. A variable high‐voltage power supply (Korea Switching Co.) was used for the electrospinning. The prepared solution was placed in a 30 mL syringe with a capillary tip (*D* = 0.5 mm). The anode of the high‐voltage power supply was clamped to a syringe needle tip, and the cathode was connected to an aluminum foil collector. The applied voltage was 20 kV, the distance between the nozzle and collector was 15 cm, and the supply rate of the solution was 1 mL h^−1^. The electrospun fibers were stabilized by heating to 280 °C at a rate of 1 °C min^−1^ in air, and holding them at that temperature for 1 h. The stabilized fibers were carbonized for 1 h at 1000 °C in nitrogen. The amounts of Fe@NGnP in PAN were 0, 3, 5, and 10 wt% (denoted as CNF‐N0, CNF‐N3, CNF‐N5, and CNF‐N10, respectively). The ratios of Fe@NGnPs to PAN were estimated using the carbonization yield of PAN‐based fibers under a nitrogen condition (31 wt%).

*Characterization and Electrochemical Measurements*: The microtexture of the nanostructured materials was examined by scanning electron microscopy (SEM, Nova FE‐SEM). The specific surface areas and pore size distributions of the samples were evaluated using the Brunauer, Emmett, and Teller (BET) method (Belsorp‐max). TEM images were obtained using a high‐ resolution TEM (JEOL, JEM‐2100F). EXAFS measurements were carried out using a Lab‐EXAFS (Rigaku, Japan) with a Ge(220) crystal. The Fe–K edge spectra were recorded in transmittance mode at room temperature. The EXAFS data were analyzed to fit the experimental EXAFS spectra to theoretical values obtained by an IFEFFIT program based on FEFF8. XPS were recorded on a Thermo Fisher Kα XPS spectrometer.

A RRDE test and a Li–air cell test were carried out using a computer‐controlled potentiostat (Biologic VMP3) with a typical three‐electrode cell. In the case of the RRDE test, a platinum wire was used as a counter‐electrode and a Hg/HgO (1 m NaOH filled) electrode was employed as a reference electrode. The working electrodes were prepared by applying each of the catalyst inks onto a pre‐polished glassy carbon disk electrode. To prepare the catalyst ink catalysts were first dispersed in an ethanol/isopropyl alcohol solution (10 mg mL^−1^) and a Nafion (25 wt%) stock solution (10 μL) in ethanol was added to the catalyst ink by bath sonication. The addition of a small amount of Nafion improved the dispersion of the catalyst suspension and enhanced the binding onto the GC electrode. A total of 5 μL of well‐dispersed catalyst ink was applied onto the prepolished glassy carbon (GC) disk electrode (5 mm in diameter). The carefully prepared electrodes were dried at room temperature before the electrochemical tests. For the Li–air cell test, a lithium foil with a thickness of 0.2 mm was obtained from Honjo Metal, and disks with a diameter of 1 cm were cut for use as the anode. 1 m LiPF_6_ in tetraethylene glycol dimethyl ether (TEGDME) was used as an organic liquid electrolyte, and 1 m LiNO_3_ + 0.5 m LiOH in pure DI water was used as the aqueous liquid electrolyte. All reagents were purchased from Sigma–Aldrich and used as received. The anode and the cathode were separated by a Li_1+*x*+*y*_Ti_2‐*x*_Al*_x_*P_3‐*y*_Si*_y_*O_12_ (0.15 mm thickness, OHARA Inc., Japan) solid Li‐ion conducting ceramic glass. The air electrodes were prepared by spraying the catalyst ink made with Pt/C, CB, and Act‐CNF‐N5 catalyst and PVdF‐HFP binder (Sigma‐Aldrich) onto the gas‐diffusion layer (Toray TGP‐H‐090). The catalyst loading density and binder content in the air electrode were 1 mg cm^−2^ and 20 wt%, respectively. Discharge–charge experiments were conducted on a Biologic VMP3 at ambient air condition.

## Supporting information

As a service to our authors and readers, this journal provides supporting information supplied by the authors. Such materials are peer reviewed and may be re‐organized for online delivery, but are not copy‐edited or typeset. Technical support issues arising from supporting information (other than missing files) should be addressed to the authors.

Supplementary
