# Supplementary material for: Fe@N‐Graphene Nanoplatelet‐Embedded Carbon Nanofibers as Efficient Electrocatalysts for Oxygen Reduction Reaction
Source: Adv Sci (Weinh). 2015 Sep 10;3(1):1500205. doi: 10.1002/advs.201500205 (PMC5049621; doi:10.1002/advs.201500205)
Supplement: Supplementary file 1 — Supplementary [file ADVS-3-0b-s001.pdf]

Copyright WILEY-VCH Verlag GmbH & Co. KGaA, 69469 Weinheim, Germany, 2015.

ADVANCED SCIENCE
Open Access

Supporting Information

for *Adv. Sci.*, DOI: 10.1002/advs.201500205

Fe@N-Graphene Nanoplatelet-Embedded Carbon Nanofibers as Efficient Electrocatalysts for Oxygen Reduction Reaction

Young-Wan Ju, Seonyoung Yoo, Changmin Kim, Seona Kim, In-Yup Jeon, Jeeyoung Shin, Jong-Beom Baek, and Guntae Kim**

Copyright WILEY-VCH Verlag GmbH & Co. KGaA, 69469 Weinheim, Germany, 2013.

Supporting Information

Fe@N-Graphene Nanoplatelets Embedded Carbon Nanofibers as Efficient Electrocatalysts for Oxygen Reduction Reaction

Young-Wan Ju, Seonyoung Yoo, Changmin Kim, Seona Kim, In-Yup Jeon, Jeeyoung Shin, Jong-Beom Baek and Guntae Kim**

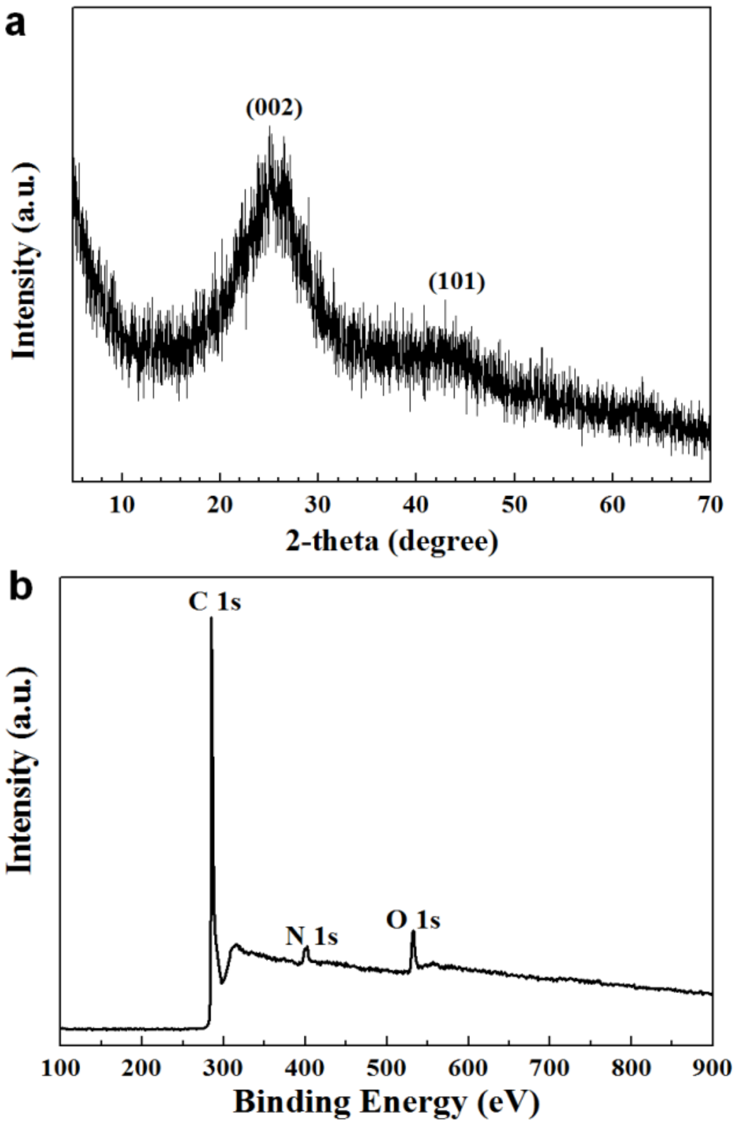

Figure S1. (a) X-ray diffraction and (b) X-ray photoelectron spectra (XPS) of N- and Fe-doped graphene nano platelet (Fe@NGnP).

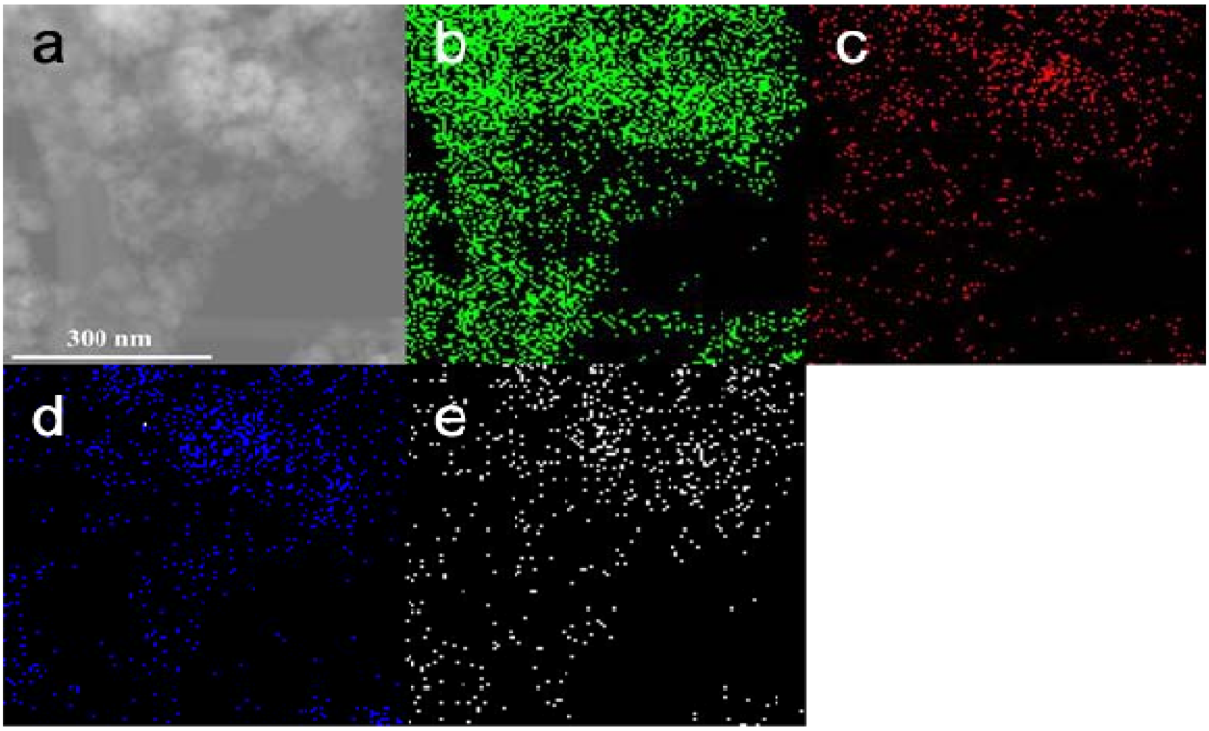

Figure S2. The Transmission Electron Microscopy (TEM) analysis: (a) Scanning TEM (STEM) high-angle annular dark-field (HAADF) image, and elemental mapping of (b) carbon, (c) nitrogen, (d) iron, and (e) oxygen, with color indicative of the signal intensity.

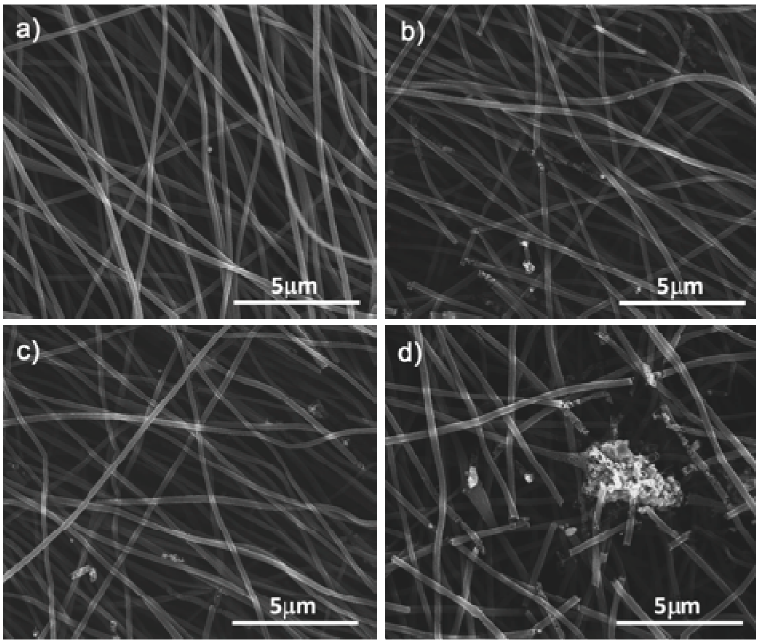

Figure S3. SEM images of carbon nano-fibers after carbonization at 1000 ^{o}C at low magnification for (a) CNF–N0, (b) CNF–N3, (c) CNF–N5, and (d) CNF–N10

Fe@NGnP-embedded carbon nanofibers(CNF) are synthesized with the as-prepared Fe@NGnP. Figure S6 exhibits SEM images of CNF(CNF-N0) and Fe@NGnP doped CNF composite (CNF-Fe@NGnP) webs carbonized at 1000 ^{o}C in N_2 atmosphere. The CNF-N0 exhibited long and continuous cylindrical morphologies, and its average diameter is approximately 220 nm, ranging from 198 to 232 nm. While the morphology of CNF-N0 (a) shows a smooth surface, the CNF-Fe@NGnP composites exhibit slenderer fiber shape, of which the average diameter is 201 nm, ranging from 188 to 220 nm, and become rough and disconnected due to the agglomeration of Fe@NGnP with increasing Fe@NGnP content. As the Fe@NGnP amount becomes 10 wt.%, the CNF-Fe@NGnP composite carbon exhibits bead-like structure (d). It is revealed that the Fe@NGnP changes in morphology of composite fiber, whereas the specific surface area of composite fiber does not vary noticeably.

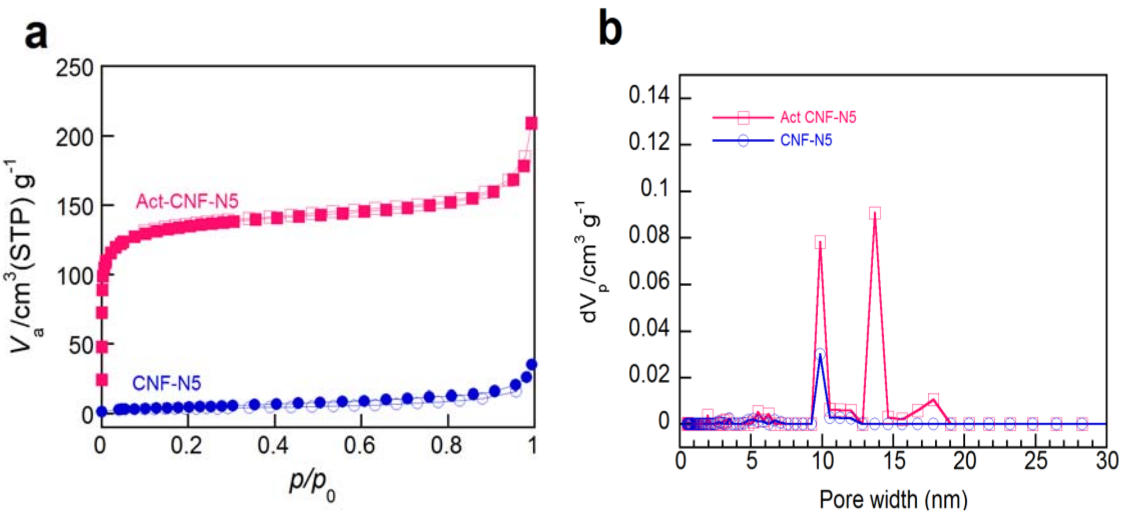

Figure S4. BET isotherms of CNF-N5 and Act-CNF-N5 (a) N_2 adsorption desorption isotherms and (b) pore size distribution curves based on the DFT method

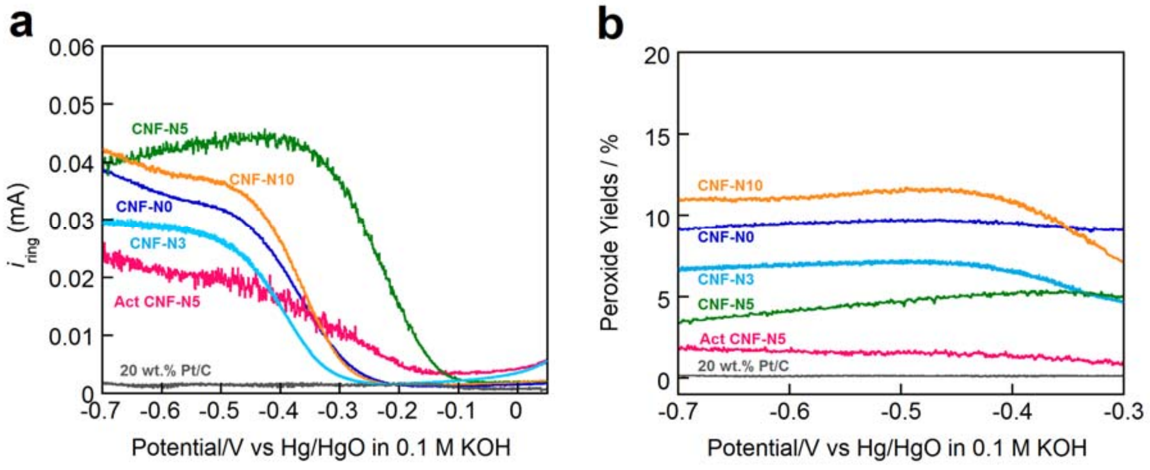

Figure S5. RRDE experiments data of ORR in 0.1 M KOH aqueous solution (scan rate = 10 mV sec^{-1}; rotation speed = 1600 rpm) (a) ring current and (b) peroxide yields (%) of as prepared samples.

The ORR catalytic activities of various CNF-Fe@NGnP composites have been investigated by the rotating ring disk electrode (RRDE) voltammograms in an O_2-saturated 0.10 M KOH aqueous solution within the potential range of -0.7 to 0.1 V. For comparison purposes, the PAN based carbon synthesized by electrospinning and the platinum (Pt) on activated carbon (Pt/C, Vulcan XC-72R) electrodes were also prepared by using the same procedure. The RRDE results show a series of polarization curves at a constant rotating rate of 1600 rpm and quantitative evaluation in terms of the onset potential and the kinetic limiting current.

To further verify the ORR activity with the optimized CNF-Fe@NGnP in comparison with those of Pt/C catalysts, the formation of hydrogen peroxide species (HO_2^-) is recorded during the ORR process by the RRDE measurements.

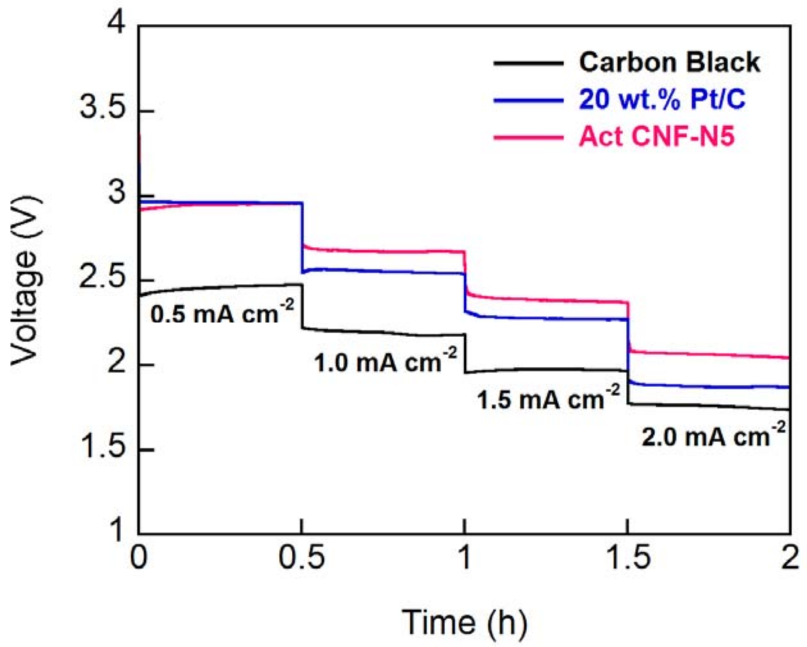

Figure S6. Discharge voltage profiles of hybrid Li-air cells with carbon black, 20 wt.% Pt/C, and Act CNF-N5 as the ORR catalyst at different current densities

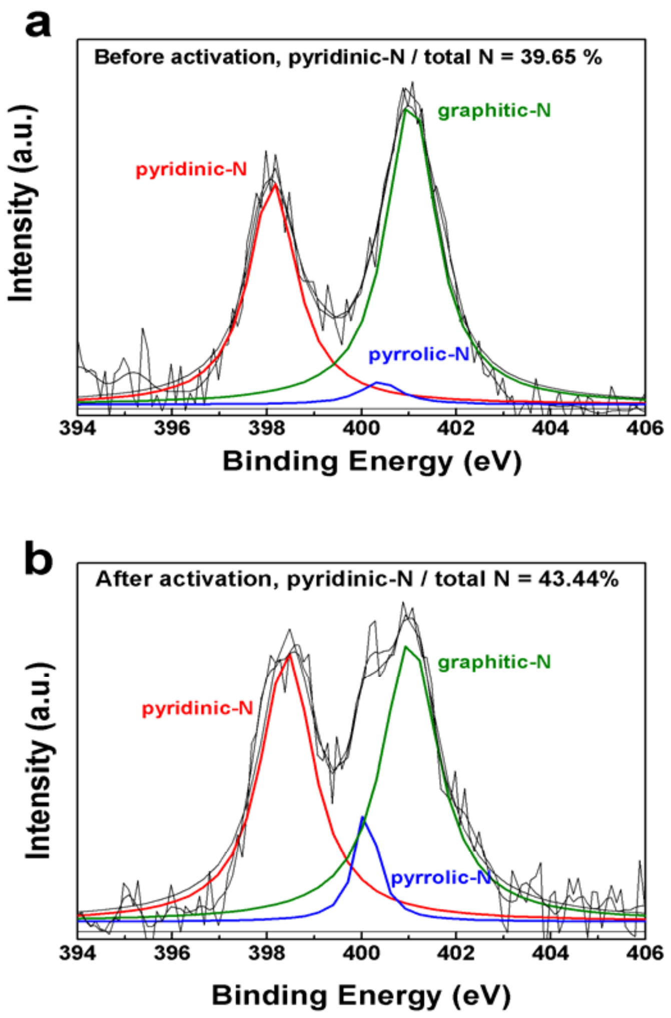

Figure S7. High resolution X-ray photoelectron spectroscopy scans for N1s of CNF–N5 (a) before activation in CO_2, and (b) after activation in CO_2.

The pyridinic-N means the nitrogen atom on the edge of graphite planes with two adjacent carbon atoms imparting Lewis basicity to the carbon. Graphite-N refers to the nitrogen atom bonded to three carbon atoms within a graphite (basal) plane, while pyrrolic-N indicates to nitrogen atoms that contribute to the system with two p-electrons. Xing *et al*. exhibited the carbon atom neighboring pyridinic-N plays an important role in ORR process and the location of pyridinic-N can affect the catalytic efficiency of carbon materials.[1] In addition, Dodelet *et al*. proposed that most of the Fe/N/C catalytic sites consist of an iron cation coordinated by four pyridinic-N attached to the edges of two graphitic sheets, each belonging to adjacent crystallites on either side of a slit pore in the carbon support.[2]

Table S1. Phase-shift corrected structural parameters obtained from the curve fitting of EXAFS spectrum of Fe@NGnPs obtained at the Fe K-edge.

		R(Å)[a]	*CN*[b]	$\Delta E_{0\,(eV)}$[c]	σ^2 ($Å^2$)[d]	R-factor
Fe	Fe-Fe	2.48	8.0	-6.72	0.003701	0.01416
	Fe-Fe	2.87	6.0			
Fe_3O_4	Fe-O	2.04	4.0	2.80	0.010741	0.00389
Fe@NGnPs -	Fe-N	1.94	2.73	1.99	0.008590	0.00029
	Fe-O	2.04	1.56	-0.56	0.004190	

[a]Interatomic distance, [b]Coordination number, [c]Energy shift. [d]Debye Waller factor

References

[1] T. Xing, Y. Zheng, L.H. Li, B.C.C. Cowie, D. Gunzelmann, S.Z. Qiao, S. Huang and Y. Chen, *ACS Nano*, **2014**, *8*, 6856-6862.

[2] M. Lefèvre, E. Proietti, F. Jaouen and J.-P. Dodelet, *Science*, **2009**, *324*, 71-74.
